# Peptide drugs accelerate BMP‐2‐induced calvarial bone regeneration and stimulate osteoblast differentiation through mTORC1 signaling

**DOI:** 10.1002/bies.201600104

**Published:** 2016-06-27

**Authors:** Yasutaka Sugamori, Setsuko Mise‐Omata, Chizuko Maeda, Shigeki Aoki, Yasuhiko Tabata, Ramachandran Murali, Hisataka Yasuda, Nobuyuki Udagawa, Hiroshi Suzuki, Masashi Honma, Kazuhiro Aoki

**Affiliations:** ^1^ Department of Bio‐Matrix (Pharmacology), Graduate School of Medical and Dental SciencesTokyo Medical and Dental UniversityTokyoJapan; ^2^ Faculty of Medicine, Department of PharmacyThe University of Tokyo Hospital, The University of TokyoTokyoJapan; ^3^ Department of Biomaterials, Field of Tissue Engineering, Institute for Frontier Medical SciencesKyoto UniversityKyotoJapan; ^4^ Research Division of Immunology, Department of Biomedical SciencesCedars‐Sinai Medical CenterLos AngelesCAUSA; ^5^ Nagahama Institute for Biochemical ScienceOriental Yeast Co. Ltd.ShigaJapan; ^6^ Department of BiochemistryMatsumoto Dental UniversityNaganoJapan; ^7^ Faculty of Medicine, Department of Pharmacology and PharmacokineticsThe University of Tokyo Hospital, The University of TokyoTokyoJapan

**Keywords:** BMP‐2, bone regeneration, histomorphometry, mTORC1, osteoblast differentiation, peptide therapeutics, rapamycin

## Abstract

Both W9 and OP3‐4 were known to bind the receptor activator of NF‐κB ligand (RANKL), inhibiting osteoclastogenesis. Recently, both peptides were shown to stimulate osteoblast differentiation; however, the mechanism underlying the activity of these peptides remains to be clarified. A primary osteoblast culture showed that rapamycin, an mTORC1 inhibitor, which was recently demonstrated to be an important serine/threonine kinase for bone formation, inhibited the peptide‐induced alkaline phosphatase activity. Furthermore, both peptides promoted the phosphorylation of Akt and S6K1, an upstream molecule of mTORC1 and the effector molecule of mTORC1, respectively. In the in vivo calvarial defect model, W9 and OP3‐4 accelerated BMP‐2‐induced bone formation to a similar extent, which was confirmed by histomorphometric analyses using fluorescence images of undecalcified sections. Our data suggest that these RANKL‐binding peptides could stimulate the mTORC1 activity, which might play a role in the acceleration of BMP‐2‐induced bone regeneration by the RANKL‐binding peptides.

## Introduction

Bone morphogenetic protein (BMP)‐2 is known to induce local bone formation and has been used clinically 1. However, recent evidence suggests that BMP‐2 has the potential to cause cancer and inflammation 2, 3, 4. In this context, a reduction in the amount of BMP‐2 that is used in BMP‐2 therapy is necessary to reduce its adverse effects 4. It is necessary to develop osteogenic agents, which can directly stimulate bone formation or at least accelerate BMP‐2‐induced bone formation.

There are several candidates for increasing local bone formation: parathyroid hormone (PTH) 5, 6, anti‐sclerostin antibody 7, transforming growth factor (TGF)‐β1 8, fibroblast growth factor (FGF)‐2 9, FGF‐4 10, and various peptide drugs 11, 12, 13, 14, 15. Among these agents, small peptide drugs, which have a molecular weight of approximately 1,000–1,500, have many advantages because of their properties, which include their low production cost, non‐immunogenic properties, and facile modulation characteristics that allow for structural changes to be made to fit a drug target 11.

WP9QY peptide (W9) was designed to mimic the critical site of binding between TNF type 1 receptor and TNF‐α 16 and was recently found to be a stimulator of BMP‐2‐induced ectopic bone formation as well as osteoblast differentiation 13, 14. OP3‐4, another peptide, which was recently demonstrated to be a systemic stimulator of bone formation in an inflammatory bone destruction model 15, was originally designed to mimic osteoprotegerin, a natural antagonist of receptor activator of NF‐κB ligand (RANKL) 17. Both W9 and OP3‐4 have been shown to bind to RANKL, a stimulator of osteoclastogenesis, thereby inhibiting osteoclast formation and bone resorption 17, 18. However, the reason why these RANKL‐binding peptides stimulate bone formation remains to be clarified 19, 20.

On the other hand, several pathways are known to stimulate osteoblast differentiation. Signaling through the BMP 21, PGE2 22, or Wnt receptors 23 eventually results in the stimulation of Runx2 expression, an early marker of osteoblast differentiation. It was recently demonstrated that rapamycin, which is an inhibitor of the mammalian target of rapamycin complex 1 (mTORC1), a serine/threonine kinase, inhibited osteoblast differentiation through the reduction of Runx2 expression in an osteoblast cell line and bone marrow‐derived stromal cells 24. Another report demonstrated that the osteoblast‐specific deletion of Raptor, one of the components of mTORC1, showed the osteopenic phenotype with a reduction of both *Col1a* and *Bglap* (osteocalcin) expression, suggesting that the mTORC1 activation could be one of the pathways for stimulating bone formation 25. Given the significant role of mTORC1 in bone formation, it has not been clarified whether the above‐described osteogenic peptides stimulate the mTORC1 activation in osteoblasts.

In the present study, we used a minimal amount of BMP‐2, which was not sufficient for inducing apparent bone formation in a murine calvarial defect model, and demonstrated that OP3‐4 stimulated BMP‐2‐induced local bone formation to the same extent as W9. We also showed that the activation of mTORC1 in OP3‐4‐treated osteoblasts was similar to that in W9‐treated osteoblasts.

## Materials and methods

### Reagents

Cyclic peptide OP3‐4 (YCEIEFCYLIR) was purchased from a peptide‐synthesizing company (Synpeptide Co., Ltd., Shanghai, China). The other cyclic peptide W9 (YCWSQYLCY) and the control peptide (FCYISEVEDECY) 17, 26 were purchased from the American Peptide Company (Sunnyvale, CA, USA). Recombinant human BMP‐2 was provided by Yamanouchi Pharmaceutical Co., Ltd. (Current Astellas Phrma Inc.; the patent has since been transferred to Bioventus LLC [Durham, NC, USA]). Gelatin hydrogel (GH; pI9) was synthesized at the department of biomaterials, field of tissue engineering, Kyoto University. The anti‐phospho‐Akt, anti‐Akt (Thr^308^), the anti‐phospho‐p70 S6 kinase, anti‐p70 S6 kinase, and anti‐GAPDH antibodies were purchased from Cell Signaling Technology (Beverly, MA, USA).

### Animals

Twenty 5‐week‐old male C57BL/6 mice were purchased from Nippon CLEA (Tokyo, Japan). The mice were maintained under normal conditions as previously described 27. All of the experimental procedures were reviewed and approved by the Animal Care and Use Committee and Recombination DNA Advisory Committee of Tokyo Medical and Dental University (Tokyo, Japan; authorization numbers: 0140070C, 0150203C2, 0160182A).

### The osteoblast proliferation and differentiation assays

In the proliferation assay, primary osteoblast‐like cells, which were isolated from the calvariae of 1‐day‐old mice were seeded at a density of 1 × 10^4^ cells/well in 96‐well plates and cultured in a proliferation medium (α‐minimum essential medium (Sigma‐Aldrich, St. Louis, MO, USA) containing 10% fetal bovine serum (Moregate Biotech, Bulimba, Australia) with penicillin (100 U/mL) and streptomycin (100 µg/mL) (Sigma‐Aldrich) for 3 days. At 72 hours after incubation, 10 µL of Cell Count Reagent SF (Nacalai Tesque, Inc., Kyoto, Japan) was added to each well. After adding the reagent, the cells were incubated for 2 hours and the optical density of each well was measured at 450 nm with a microplate reader (Model 550 Microplate Reader, Bio‐Rad, Hercules, CA, USA).

In the differentiation assay, primary osteoblasts isolated from the calvariae of 1‐day‐old mice were seeded at a density of 5 × 10^4^ cells/well in 24‐well plates and cultured in a differentiation medium, α‐minimum essential medium containing 10% fetal bovine serum, 50 μg/mL of ascorbic acid (Wako, Osaka, Japan), 10 mM β‐glycerophosphate (Sigma‐Aldrich), 10 nM dexamethasone (Sigma‐Aldrich), 100 U/mL penicillin, and 100 µg/mL streptomycin (Sigma‐Aldrich). The conditioned medium was changed every 3 days. Alkaline phosphatase (ALP) staining and von Kossa staining were performed on days 6 and 21, respectively, according to standard protocols 28. Each positively stained area was measured with an image analyzing system and calculated as a percentage of the whole well area (KS 400; Carl Zeiss, Jena, Germany). In some cultures, ALP activity was measured as follows. The primary osteoblast‐like cells were seeded at a density of 1 × 10^4^ cells/well in 96‐well plates and cultured in the above‐described differentiation medium for 6 days. The medium was changed on day 3. On day 6, 1% NP‐40 and ALP solution, including 1 mg/mL p‐nitrophenyl phosphate (pNPP) (Sigma‐Aldrich, MO, USA) and 1 mM MgCl_2_, 0.2 M Tri‐HCl (pH 9.5) were added to each well and incubated for 10 minutes at 37°C. The ALP activity in each well was measured at 415 nm with a microplate reader (Model 550 Microplate Reader, Bio‐Rad).

### The experimental design of calvarial defect model

Twenty 5‐week‐old male C57BL/6 mice were divided into four groups. Vehicle, BMP‐2 (0.3 µg), BMP‐2 (0.3 µg) plus W9 (0.4 µmol) or BMP‐2 (0.3 µg) plus OP3‐4 (0.4 µmol) were incorporated in gelatin hydrogel (GH) discs. The GH containing each agent(s) was placed on a defect of 3.5 mm in diameter that had been made on the right parietal bone using a biopsy punch (Kai Industries, Gifu, Japan), as described elsewhere 12. All mice were injected with calcein and alizarin red on days 15 and 20, respectively. They were sacrificed on day 28 after the operation.

### Radiographic analyses

Soft X‐ray photographs of the calvariae were taken with an X‐ray device (SRO‐M50; Sofron, Tokyo, Japan). Three‐dimensional (3D) reconstruction images of the calvariae were captured by microfocal computed tomography (μCT) (Scan Xmate‐E090; Comscan, Yokohama, Japan). The bone mineral content and bone mineral density of the calvariae were measured by dual‐energy X‐ray absorptiometry (DXA) (DCS‐600 R; Aloka, Tokyo, Japan).

### Histological preparation and bone histomorphometry

Undecalcified frozen sections of 5 μm in thickness were prepared as described elsewhere 29. Bone histomorphometry was performed in a rectangular ROI (2.24 × 3.85 mm^2^) at the center of the defect site. The mineralizing surface (MS) and mineral apposition rate (MAR) were measured using a KS400 image analyzing system, as previously described 15, 30. Local bone formation activity was defined as MS × MAR.

### mTORC1 signaling assay

ST‐2 cells (a murine osteoblastic cell line [RIKEN, Ibaragi, Japan]), were maintained in α‐minimum essential medium (Sigma‐Aldrich) containing 10% fetal bovine serum (Moregate Biotech), penicillin (100 U/mL) and streptomycin (100 µg/mL) (Sigma‐Aldrich) 31, 32. After 12 hours of serum starvation, the cells were treated with vehicle or various concentrations of W9 or OP3‐4, as indicated, for 20 minutes. Total cell lysates were prepared and analyzed by Western blotting as described previously 31, 32. Briefly, ice‐cold PBS was used to stop the stimulation and the cells were immediately lysed lysis buffer (PBS, pH 7.4, 1.0% Triton X‐100) supplemented with protease inhibitors (Roche Diagnostics, Indianapolis, IN, USA) and phosphatase inhibitors (Sigma‐Aldrich). Western blotting was performed to evaluate Akt and S6K1 phosphorylation. Anti‐phospho‐Akt, anti‐Akt, anti‐phospho‐p70 S6 kinase, anti‐p70 S6 kinase, and anti‐GAPDH antibodies were used as the primary antibodies (Cell Signaling Technology). Proteins were then detected with an HRP‐labeled secondary antibody (GE Healthcare, Buckinghamshire, UK), ECL Prime reagent (GE Healthcare Bioscience) using a Chemidoc XRS (Bio‐Rad).

### Statistical analyses

All of the data were expressed as means ± SD. For both the in vivo and in vitro studies, the statistically significant differences among the groups were assessed by a one‐way analysis of variance (ANOVA). When significant *F*‐values were detected, Fisher's PLSD post hoc test was performed. *p*‐Values of <0.05 were considered to indicate statistical significance.

## Results

### OP3‐4 promoted osteoblast differentiation to the same extent as W9

**Figure 1:**
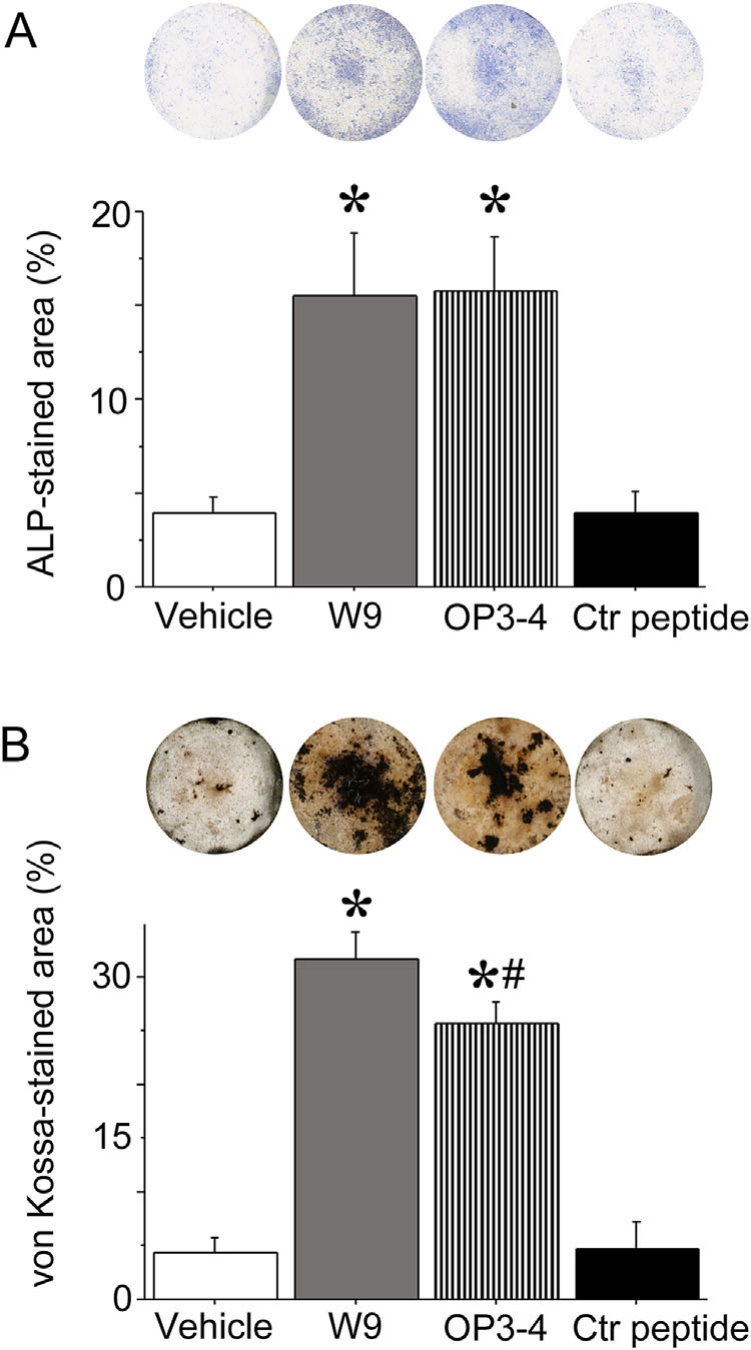
RANKL‐binding peptides, W9 and OP3‐4, enhanced ALP activity and mineralization during osteoblast differentiation. **A:** Primary osteoblasts were cultured in differentiation medium for 6 days in the presence of RANKL‐binding peptides or a control peptide at 200 µM. The osteoblasts were fixed and ALP staining was performed. Images of the ALP‐stained cells are shown. The percentage of the ALP‐stained area in each well was calculated. **B:** Cells were cultured for 21 days and von Kossa staining was performed. Images of the von Kossa‐stained cells are shown. The percentage of the von Kossa‐stained area in each well was calculated. The data are expressed as the means ± standard deviation for each group (*n* = 5). Significant differences among the groups were assessed by ANOVA. When significant *F*‐values were detected, then Fisher's PLSD post hoc test was performed. *p < 0.05 versus vehicle, #*p* < 0.05 versus W9. We performed three‐independent experiments and obtained similar results. The data are the representative results of the three experiments.

First we compared the osteogenic activity of W9 and OP3‐4 in an osteoblast differentiation assay in vitro. As shown in Fig. 1A and B, OP3‐4 promoted increases in the ALP‐ and von Kossa‐stained areas, which are indices of early and late osteoblast differentiation, respectively, to the same extent as W9. Quantitative measurements confirmed these observations.

### OP3‐4 showed similar bone regeneration to W9 in the murine calvarial defect model, with a minimal amount of BMP‐2

**Figure 2:**
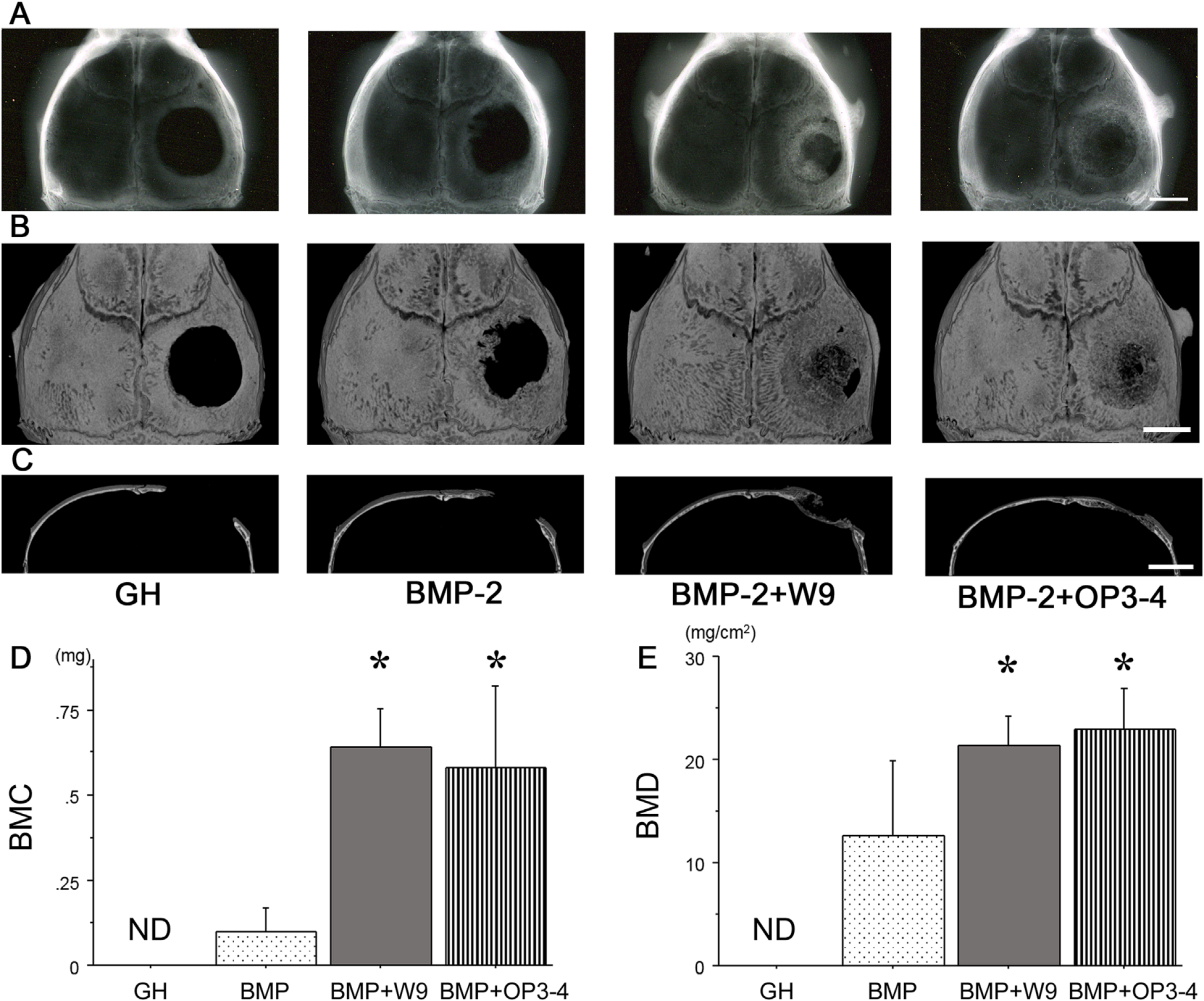
RANKL‐binding peptides, W9 and OP3‐4, promoted bone regeneration induced by BMP‐2 in a murine calvarial defect model. **A:** Soft X‐ray photographic images of the calvarial defects in which gelatin hydrogel (GH) only, and GH containing BMP‐2 (0.3 µg), BMP‐2 (0.3 µg) plus W9 (0.4 µmol), or BMP‐2 (0.3 µg) plus OP3‐4 (0.4 µmol) were applied. **B** and **C:** µ‐CT images of the whole mount of calvariae (**B**) and a cross‐section (**C**) are shown for each group. Scale bars: 2 mm. **D** and **E:** The bone mineral content (BMC) and bone mineral density (BMD) were measured at the site of the calvarial defect using dual energy X‐ray absorptiometry. The data are expressed as the means ± standard deviation for each group (*n* = 5). Significant differences among the groups were assessed by ANOVA. When significant *F*‐values were detected, then Fisher's PLSD post hoc test was performed. *p < 0.05 versus BMP group. We performed two‐independent experiments and obtained similar results. The data are the representative results of the two experiments.

A murine calvarial defect model was used to clarify whether OP3‐4 stimulates bone regeneration in vivo to the same extent as W9. The size of the defect was a critical size that would preclude the natural recovery of bone in 4 weeks. In our preliminary experiment, we determined the amount of BMP‐2 that could be applied without showing apparent bone regeneration in the model. The mice were sacrificed 4 weeks after the placement of GH carrier alone, or GH containing BMP‐2 with or without a peptide, on the calvarial defect. First the local bone formation was radiologically analyzed. Soft X‐ray and μCT images revealed a very small radio‐opaque area at the bone defect site in the group that received the GH carrier with BMP‐2; the size appeared to be almost the same as that in the carrier alone group. On the other hand, the radio‐opaque area was greatly enhanced when GH containing BMP‐2 and a peptide was applied. The radio‐opaque area in the BMP and OP3‐4 group appeared to be the same as that in the BMP‐2 and W9 group (Fig. 2A–C). A quantitative analysis of the bone mineral content (BMC) and bone mineral density (BMD) at the defect site confirmed these observations (Fig. 2D and E). The treatment of the RANKL‐binding peptide without BMP‐2 did not show any apparent increase of bone formation in vivo (Supplementary Fig. S1).

### Both W9 and OP3‐4 stimulated bone formation activity at the site of the calvarial defect

**Figure 3:**
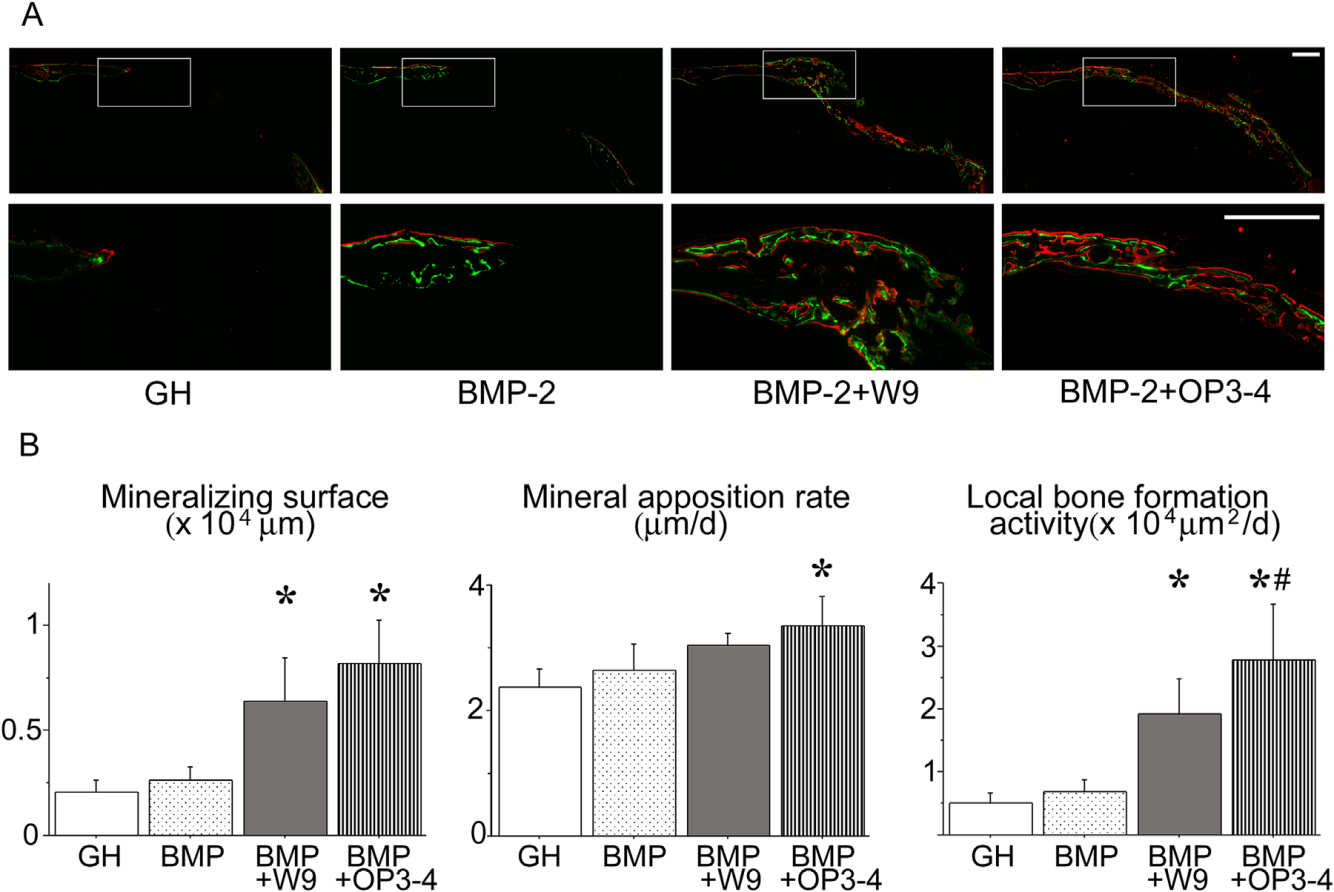
RANKL‐binding peptides, W9 and OP3‐4, promoted mineralization in vivo. **A:** Fluorescence images of undecalcified frozen sections of the right side of the calvaria are shown. The lower panels indicate higher magnification views of the box shown in white in the upper panels. The scale bar represents 0.5 mm. The green color shows calcein labeling, while the red color shows the alizarin labeling. **B:** Quantitative analyses of bone formation activity were performed using standard bone histomorphometric measurement techniques based on the calcein‐ and alizarin red‐labeled surface in the ROI (as described in Materials and methods section), centering on the area of regenerated bone. Local bone formation activity was calculated as (mineralizing surface) × (mineral apposition rate). The data are expressed as the means ± standard deviation for each group (*n* = 5). Significant differences among the groups were assessed by ANOVA. When significant *F*‐values were detected, then Fisher's PLSD post hoc test was performed.*p < 0.05 versus BMP, #*p* < 0.05 versus BMP + W9. We performed two‐independent experiments and obtained similar results. The data are the representative results of the two experiments.

Fluorescence images of undecalcified sections revealed bone formation activity at the site of the calvarial defect. The calcein‐ and alizarin‐labeled surface seemed to slightly increase in the BMP‐2 group compared to the GH carrier group and the surface appeared to increase in the peptide applied groups compared to both the GH carrier group and the BMP‐2 group (Fig. 3A). Quantitative analyses revealed that a significant increase of the mineralizing surface, which reflects the number of active osteoblasts, in the peptide applied groups compared to the GH carrier group and BMP‐2 group (Fig. 3B). Local bone formation activity, which indicates the total calcified area that is produced in the region of interest in a single day, also showed similar results to those of the mineralizing surface (Fig. 3B). To clarify the contribution of the RANKL‐binding peptides as a bone resorption inhibitor, we counted the number of osteoclasts that appeared at the bone regeneration site of each group. The number of osteoclasts in both the BMP‐2 + W9 group and the BMP‐2 + OP3‐4 group actually increased compared to the BMP‐2 group as shown in Supplementary Fig. S2.

### Rapamycin, an mTORC1 inhibitor, inhibited the peptide‐induced osteoblast differentiation

**Figure 4:**
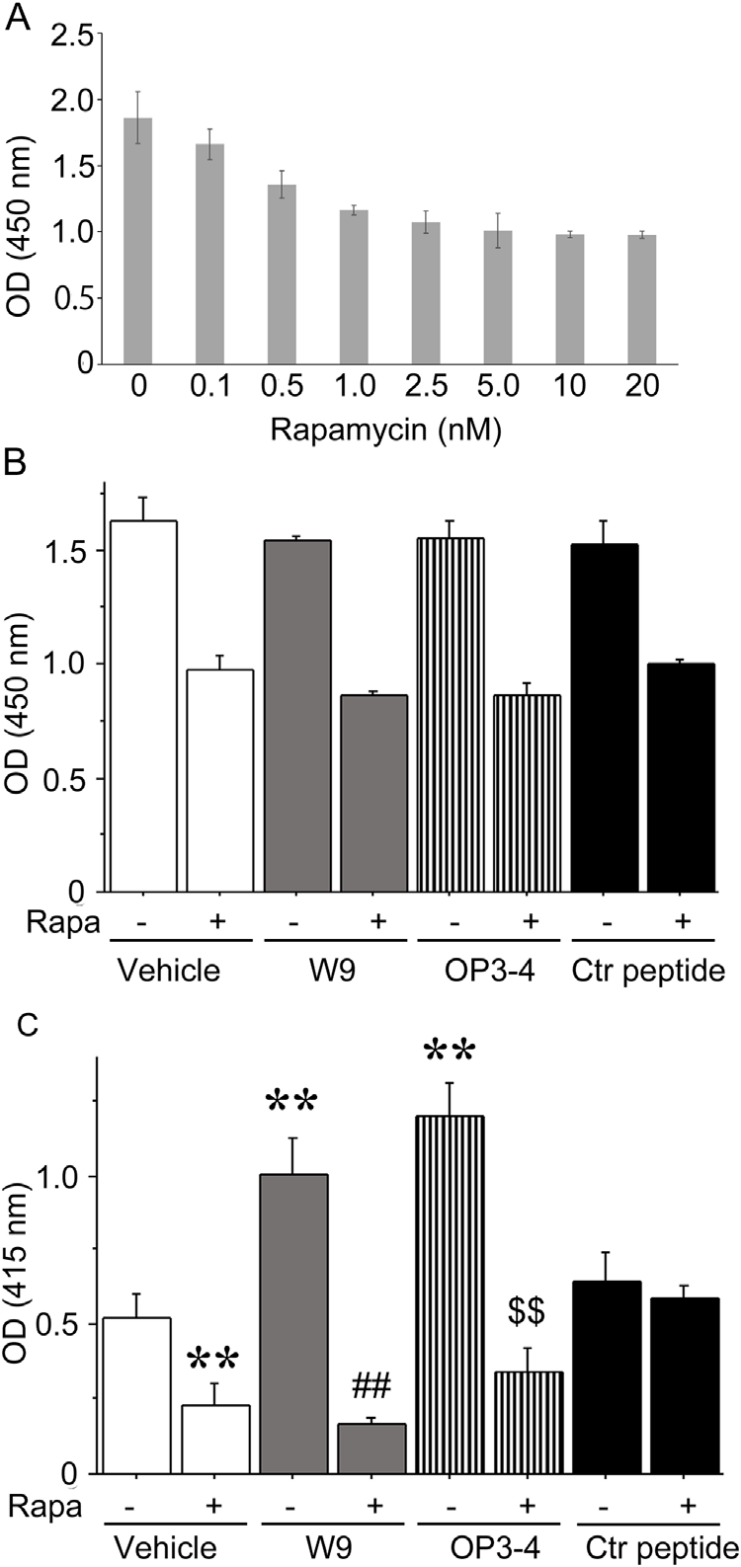
mTORC1 inhibitor blunted the stimulated effects of osteoblast differentiation by RANKL‐binding peptides. **A:** Rapamycin inhibits osteoblast proliferation. The indicated concentration of rapamycin (0.1–20 nM) was added to cultures of primary osteoblasts in proliferation medium. Cell proliferation was assayed for 72 hours using Cell Count Reagent SF. **B:** RANKL‐binding peptides did not affect osteoblast proliferation. Primary osteoblasts were cultured with vehicle, W9, OP3‐4, or the control peptide in the presence or absence of rapamycin (10 nM), and cell proliferation was assayed. **C:** Rapamycin inhibited osteoblast differentiation. The differentiation of primary osteoblasts was induced during 6 days of culturing in differentiation medium in the absence or presence of rapamycin (Rapa) (10 nM) and ALP activity was measured. The data are expressed as the means ± standard deviation for each group (*n* = 5). Significant differences among the groups were assessed by ANOVA. When significant *F*‐values were detected, then Fisher's PLSD post hoc test was performed. **p < 0.01 versus vehicle without Rapa, ^##^ *p* < 0.01 versus W9 without Rapa, $$*p* < 0.01 versus OP3‐4 without Rapa. We performed two‐independent experiments and obtained similar results. The data are the representative results of the two experiments.

Since mTORC1 signaling has been shown to be one of the important signaling targets for bone formation 24, 25, we investigated whether rapamycin, an inhibitor of mTORC1 activity, could reduce the peptide‐induced acceleration of ALP activity, one of the markers of osteoblast differentiation. Before starting this experiment, we first determined a dose of rapamycin which would not show cytotoxicity in the osteoblast cultures. A proliferation assay using primary osteoblasts, which were isolated from the calvariae of new born mice, was performed in the presence of various concentrations of rapamycin. At low concentrations of rapamycin (0–5 nM), cell proliferation gradually decreased in a dose‐dependent manner, but it gradually plateaued at high concentrations (5–20 nM), suggesting that a dose of less than 20 nM of rapamycin might not affect cell viability (Fig. 4A).

We next evaluated the effects of W9 and OP3‐4 on osteoblast proliferation and the involvement of mTORC1 activity in cellular proliferation using 10 nM rapamycin. Figure 4B showed that W9 and OP3‐4 did not affect the proliferation of osteoblasts. Rapamycin suppressed cellular proliferation at an equal level, regardless of the presence of either W9 or OP3‐4. These results indicated that mTORC1 activity was involved in cellular proliferation; however, these two peptides did not affect osteoblast proliferation (Fig. 4B).

In order to clarify the involvement of mTORC1 activity in osteoblast differentiation, we examined the effect of rapamycin on the peptide‐induced acceleration of osteoblast differentiation in vitro. Both peptides enhanced the ALP activity as shown in Fig. 1A and the presence of rapamycin blunted the stimulation that was caused by the peptides (Fig. 4C). These results suggest that mTORC1 activity is involved in RANKL‐binding peptides‐mediated osteoblast differentiation.

### OP3‐4 activated signaling through mTORC1 to the same extent as W9

**Figure 5:**
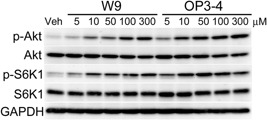
Both W9 and OP3‐4 enhanced the phosphorylation of Akt and S6K1, a downstream effector molecule of mTORC1 in the same manner. ST‐2 cells were seeded with 4 × 10^4^ cells/ per well in a 12‐well‐plate. The cells were stimulated by several concentrations of either W9 or OP3‐4 for 20 minutes after serum‐starvation for 12 hours, and then the cells were extracted and prepared for Western blotting to observe the phosphorylation of Akt and S6K1, which shows the upstream kinase of mTORC1 signals and the mTORC1 signal, respectively. We performed two‐independent experiments and obtained similar results. The data are the representative results of the two experiments.

In order to clarify whether W9 or OP3‐4 stimulates the mTORC1 pathway, we examined the phosphorylation of Akt, an upstream molecule of mTORC1 and the phosphorylation of S6K1, a downstream effector molecule of mTORC1 after stimulation with various concentrations of each peptide. OP3‐4 enhanced the phosphorylation of both Akt and S6K1 to the same extent as W9, indicating that both peptides activate mTORC1 signaling (Fig. 5).

## Discussion

In the present study, we set the amount of BMP‐2 at a level for which bone regeneration could not be observed in our calvarial defect model. This was not because the BMP‐2 used in this study was unable to regenerate bone – as 1 μg of the same BMP‐2 was shown to cover the entire calvarial defect in the same murine model 12. We demonstrated that the RANKL‐binding peptide OP3‐4 stimulated the BMP‐2‐induced bone formation to the same extent as another RANKL‐binding peptide, W9, which is already known to be a stimulator of bone formation 12, 13, 14 (Figs 2 and 3). However, the RANKL‐binding peptides seem to induce osteogenic effects only in the presence of BMP‐2 in vivo since no apparent bone formation appeared after administering the RANKL‐binding peptides in this study (Supplementary Fig. S1). We also showed that the mechanism under which the both RANKL‐binding peptides stimulated bone formation could be explained by the activation of mTORC1 signaling, since rapamycin, an mTORC1 inhibitor, is known to inhibit Runx2 activation and ALP expression 24 (Figs 4 and 5).

As described in the Introduction section, mTORC1 inhibition by rapamycin was shown to reduce the proliferation of the MC3T3E1 murine osteoblastic cell line and murine bone marrow stromal cells 24. Our data showed that the RANKL‐binding peptides did not stimulate the proliferation of primary osteoblastic cells and that proliferation was inhibited by rapamycin (an mTORC1 inhibitor) to a similar extent in all of the groups (Fig. 4B). This suggested that our peptides were not involved in the enhancement of mature osteoblast proliferation. Since the mesenchymal stem cells of muscle tissue, which are known as satellite cells, have been shown to stimulate proliferation through mTORC1 signaling 33, our peptide might also stimulate the proliferation of early precursors of osteoblasts and the differentiation of osteoblasts through the stimulation of the mTORC1 pathway (Fig. 5). Further studies are necessary to clarify whether the RANKL‐binding peptides stimulate the proliferation of cells related to bone formation.

We showed that the inhibition of mTORC1 using rapamycin led to a reduction of the RANKL‐binding peptide‐induced osteoblast differentiation. Although this inhibition included a reduction in proliferation, the inhibition of osteoblast differentiation by the RANKL‐binding peptides was far greater than the reduction of proliferation that was shown in Fig. 4B, suggesting that mTORC1 signaling was involved in the stimulation of osteoblast differentiation. Furthermore, this inhibition was not due to cytotoxicity or the cell death of primary osteoblasts because the proliferation level of the primary osteoblasts was similar, even after administering a double concentration of rapamycin, compared to that used in Fig. 4C. Taken together, our data showed, for the first time, that W9 and OP3‐4, the RANKL‐binding peptides, increased osteoblast differentiation through mTORC1 signaling, which was confirmed by the increased the phosphorylation of Akt, the upstream kinase of mTORC1 signals, and the phosphorylation of S6K1, the effector molecule of mTORC1, by the RANKL‐binding peptides (Fig. 5).

We have previously reported that the stimulated effects of W9 on the ALP activity, one of the osteoblast differentiation markers, decreased when using cells where RANKL was either knocked down or deleted, thereby indicating the existence of RANKL‐reverse signals 13. Given that OP3‐4 binds to RANKL to a similar extent as that to W9 17, 18, these data suggest that both W9 and OP3‐4 stimulate osteoblast differentiation through membrane‐bound RANKL in osteoblasts. Additional experiments showing that a mutation in the intracellular domain of RANKL reduces the phosphorylation of Akt and S6K1 would therefore be necessary to confirm the existence of the RANKL‐binding peptide‐induced RANKL‐reverse signals. Further study may be necessary regarding the reverse signals using ST‐2 cells since the RANKL expressions are very low on ST‐2 cells in the steady state. Kariya et al. 34 have already shown that RANKL is predominantly localized in lysosomes in the steady state and is translocated into the plasma membranes after stimulation with RANKL using RANK‐Fc‐conjugated beads in ST‐2 cells. Our preliminary experiment also showed that GFP‐RANKL, which was transfected in ST‐2 cells, is translocated from lysosomes to the plasma membrane within 2 hours after the stimulation with the RANKL‐binding peptide, W9 (data not shown). Thus, we considered that sufficient amount of membrane‐bound RANKL to activate the cells could accumulate at the plasma membranes, and the RANKL‐binding peptides could thus stimulate the phosphorylation of Akt and S6K1 as shown in Fig. 5.

Considering the relevance of RANKL and BMP‐2 in this study, some crosstalk might exist between the BMP‐2 signals and the RANKL‐mediated signals since we have previously shown that the RANKL‐binding peptide‐induced increase of ALP activity was partially blocked by adding the neutralizing antibody of BMP‐2/4 in the 5 days culture of an osteoblast cell line 13. In addition, there is some activation of phosphorylation of Smad 1/5/8 at 30 and 60 minutes after the W9 stimulation 13. Furthermore, the RANKL‐binding peptide could stimulate the BMP‐2‐induced increase of the ALP activity and mineralization in MC3T3E1 cells and primary osteoblasts 13, 14. Therefore, our data suggest that BMP‐2 signals and RANKL‐mediated signals synergistically stimulated each other and thereby stimulated BMP‐2‐induced osteogenesis in the calvarial defect model in vivo.

When the number of osteoclasts at the bone regeneration site was measured, it was found to increase in both the BMP‐2 + W9 group and the BMP‐2 + OP3‐4 group (Supplementary Fig. S2), although W9 and OP3‐4 are reported to inhibit the osteoclast differentiation induced by RANKL 17, 18. Considering the life span of murine osteoclasts, which is generally believed to be around 3–4 days in vivo, these data on osteoclasts suggest that the peptide‐release from the gelatin hydrogel carrier did not take place in the last phase of the experiment. Therefore, we concluded that the contribution of the peptide as a bone resorption inhibitor on the increase of BMC and BMD was therefore less than its contribution as a bone formation stimulator.

The size of the defect used in this study was considered to be a critical size since it would take around 7 months to repair the whole defect of the calvaria based on the calculation from the local bone formation activity although the previous literature show that a 4 mm diameter could be a marginal size of the defect 35, 36. This calculation was performed as shown below; the local bone formation activity, which shows the calcified bone area made in a day, was around 5 × 10^3^ µm^2^ in the GH carrier group as shown in Fig. 3B and the size of the defect was approximately 105 × 10^3^ µm^2^ (3.5 × 0.3 mm^2^ in size). When the defect bone size was divided by the bone formation activity of the GH carrier group, the expected repair time was thus obtained. Taken together the defect size we made in this study it thus considered to be a critical size defect.

In conclusion, the RANKL‐binding peptides stimulated BMP‐2‐induced bone formation, even when a minimal amount of BMP‐2 was applied. This augmentation of local bone formation could be due to the acceleration of osteoblast differentiation by the RANKL‐binding peptides, which were involved in the activation of mTORC1 signaling.

The authors have declared no conflicts of interest.

## Supporting information

As a service to our authors and readers, this journal provides supporting information supplied by the authors. Such materials are peer reviewed and may be re‐organized for online delivery, but are not copy‐edited or typeset. Technical support issues arising from supporting information (other than missing files) should be addressed to the authors.

**Fig. S1**. RANKL‐binding peptides without BMP‐2 did not promote bone regeneration in a murine calvarial defect model.

**Fig. S2**. Bone resorption parameters at the bone regeneration site.

Supplementary Figure Legends

## References

[1] McKay WF , Peckham SM , Badura JM. 2007 A comprehensive clinical review of recombinant human bone morphogenetic protein‐2 (INFUSE Bone Graft). Int Orthop 31: 729–34. 17639384 10.1007/s00264-007-0418-6 PMC2266665

[2] Feeley BT , Krenek L , Liu N , Hsu WK , et al. 2006 Overexpression of noggin inhibits BMP‐mediated growth of osteolytic prostate cancer lesions. Bone 38: 154–66. 16126463 10.1016/j.bone.2005.07.015

[3] Woo EJ. 2013 Adverse events after recombinant human BMP2 in nonspinal orthopaedic procedures. Clin Orthop Relat Res 471: 1707–11. 23132207 10.1007/s11999-012-2684-x PMC3613534

[4] Carreira AC , Lojudice FH , Halcsik E , Navarro RD , et al. 2014 Bone morphogenetic proteins: facts, challenges, and future perspectives. J Dent Res 93: 335–45. 24389809 10.1177/0022034513518561

[5] Yun JI , Wikesjo UM , Borke JL , Bisch FC , et al. 2010 Effect of systemic parathyroid hormone (1‐34) and a beta‐tricalcium phosphate biomaterial on local bone formation in a critical‐size rat calvarial defect model. J Clin Periodontol 37: 419–26. 20236187 10.1111/j.1600-051X.2010.01547.x

[6] Kuroshima S , Kovacic BL , Kozloff KM , McCauley LK , et al. 2013 Intra‐oral PTH administration promotes tooth extraction socket healing. J Dent Res 92: 553–9. 23611925 10.1177/0022034513487558 PMC3654759

[7] Han P , Ivanovski S , Crawford R , Xiao Y. 2015 Activation of the canonical Wnt signaling pathway induces cementum regeneration. J Bone Miner Res 30: 1160–74. 25556853 10.1002/jbmr.2445

[8] Tachi K , Takami M , Sato H , Mochizuki A , et al. 2011 Enhancement of bone morphogenetic protein‐2‐induced ectopic bone formation by transforming growth factor‐beta1. Tissue Eng Part A 17: 597–606. 20874259 10.1089/ten.TEA.2010.0094

[9] Nakamura Y , Tensho K , Nakaya H , Nawata M , et al. 2005 Low dose fibroblast growth factor‐2 (FGF‐2) enhances bone morphogenetic protein‐2 (BMP‐2)‐induced ectopic bone formation in mice. Bone 36: 399–407. 15777655 10.1016/j.bone.2004.11.010

[10] Kubota K , Iseki S , Kuroda S , Oida S , et al. 2002 Synergistic effect of fibroblast growth factor‐4 in ectopic bone formation induced by bone morphogenetic protein‐2. Bone 31: 465–71. 12398941 10.1016/s8756-3282(02)00852-9

[11] Aoki K , Alles N , Soysa N , Ohya K. 2012 Peptide‐based delivery to bone. Adv Drug Deliv Rev 64: 1220–38. 22709649 10.1016/j.addr.2012.05.017

[12] Al Mamun MA , Khan MAAM , Alles N , Matsui M , et al. 2013 Gelatin hydrogel carrier with the W9‐peptide elicits synergistic effects on BMP‐2‐induced bone regeneration. J Oral Biosci 55: 217–23.

[13] Furuya Y , Inagaki A , Khan M , Mori K , et al. 2013 Stimulation of bone formation in cortical bone of mice treated with a receptor activator of nuclear factor‐kappaB ligand (RANKL)‐binding peptide that possesses osteoclastogenesis inhibitory activity. J Biol Chem 288: 5562–71. 23319583 10.1074/jbc.M112.426080 PMC3581422

[14] Masud Khan AA , Alles N , Soysa NS , Al Mamun MA , et al. 2013 The local administration of TNF‐α and RANKL antagonist peptide promotes BMP‐2‐induced bone formation. J Oral Biosci 55: 47–54.

[15] Kato G , Shimizu Y , Arai Y , Suzuki N , et al. 2015 The inhibitory effects of a RANKL‐binding peptide on articular and periarticular bone loss in a murine model of collagen‐induced arthritis: a bone histomorphometric study. Arthritis Res Ther 17: 251. 26373710 10.1186/s13075-015-0753-8 PMC4570694

[16] Takasaki W , Kajino Y , Kajino K , Murali R , et al. 1997 Structure‐based design and characterization of exocyclic peptidomimetics that inhibit TNF alpha binding to its receptor. Nat Biotechnol 15: 1266–70. 9359109 10.1038/nbt1197-1266

[17] Cheng X , Kinosaki M , Takami M , Choi Y , et al. 2004 Disabling of receptor activator of nuclear factor‐kappaB (RANK) receptor complex by novel osteoprotegerin‐like peptidomimetics restores bone loss in vivo. J Biol Chem 279: 8269–77. 14679212 10.1074/jbc.M309690200

[18] Aoki K , Saito H , Itzstein C , Ishiguro M , et al. 2006 A TNF receptor loop peptide mimic blocks RANK ligand‐induced signaling, bone resorption, and bone loss. J Clin Invest 116: 1525–34. 16680194 10.1172/JCI22513 PMC1448165

[19] Henriksen K , Karsdal MA , Martin TJ. 2014 Osteoclast‐derived coupling factors in bone remodeling. Calcif Tissue Int 94: 88–97. 23700149 10.1007/s00223-013-9741-7

[20] Sims NA , Romas E. 2015 Is RANKL inhibition both anti‐resorptive and anabolic in rheumatoid arthritis? Arthritis Res Ther 17: 328. 26577945 10.1186/s13075-015-0861-5 PMC4650503

[21] Komori T. 2005 Regulation of skeletal development by the Runx family of transcription factors. J Cell Biochem 95: 445–53. 15786491 10.1002/jcb.20420

[22] Yoshida K , Oida H , Kobayashi T , Maruyama T , et al. 2002 Stimulation of bone formation and prevention of bone loss by prostaglandin E EP4 receptor activation. Proc Natl Acad Sci USA 99: 4580–5. 11917107 10.1073/pnas.062053399 PMC123690

[23] Rodriguez‐Carballo E , Ulsamer A , Susperregui AR , Manzanares‐Cespedes C , et al. 2011 Conserved regulatory motifs in osteogenic gene promoters integrate cooperative effects of canonical Wnt and BMP pathways. J Bone Miner Res 26: 718–29. 20878775 10.1002/jbmr.260

[24] Singha UK , Jiang Y , Yu S , Luo M , et al. 2008 Rapamycin inhibits osteoblast proliferation and differentiation in MC3T3‐E1 cells and primary mouse bone marrow stromal cells. J Cell Biochem 103: 434–46. 17516572 10.1002/jcb.21411

[25] Chen J , Long F. 2015 mTORC1 signaling promotes osteoblast differentiation from preosteoblasts. PLoS ONE 10: e0130627. 26090674 10.1371/journal.pone.0130627 PMC4474698

[26] Heath DJ , Vanderkerken K , Cheng X , Gallagher O , et al. 2007 An osteoprotegerin‐like peptidomimetic inhibits osteoclastic bone resorption and osteolytic bone disease in myeloma. Cancer Res 67: 202–8. 17210700 10.1158/0008-5472.CAN-06-1287

[27] Saito H , Kojima T , Takahashi M , Horne WC , et al. 2007 A tumor necrosis factor receptor loop peptide mimic inhibits bone destruction to the same extent as anti‐tumor necrosis factor monoclonal antibody in murine collagen‐induced arthritis. Arthritis Rheum 56: 1164–74. 17393436 10.1002/art.22495

[28] Recker RR. 1983 In Recker RR, ed; Bone Histomorphometry: Techniques and Interpretation. Boca Raton, Florida: CRC Press. p. 23, p. 32.

[29] Suzuki N , Aoki K , Marcian P , Borak L , et al. 2015 A threshold of mechanical strain intensity for the direct activation of osteoblast function exists in a murine maxilla loading model. Biomech Model Mechanobiol, Epub ahead of print, doi: 10.1007/s10237‐015‐0746‐1 10.1007/s10237-015-0746-1 26578077

[30] Dempster DW , Compston JE , Drezner MK , Glorieux FH , et al. 2013 Standardized nomenclature, symbols, and units for bone histomorphometry: a 2012 update of the report of the ASBMR Histomorphometry Nomenclature Committee. J Bone Miner Res 28: 2–17. 23197339 10.1002/jbmr.1805 PMC3672237

[31] Aoki S , Honma M , Kariya Y , Nakamichi Y , et al. 2010 Function of OPG as a traffic regulator for RANKL is crucial for controlled osteoclastogenesis. J Bone Miner Res 25: 1907–21. 20560139 10.1002/jbmr.89

[32] Honma M , Ikebuchi Y , Kariya Y , Hayashi M , et al. 2013 RANKL subcellular trafficking and regulatory mechanisms in osteocytes. J Bone Miner Res 28: 1936–49. 23529793 10.1002/jbmr.1941

[33] Rodgers JT , King KY , Brett JO , Cromie MJ , et al. 2014 mTORC1 controls the adaptive transition of quiescent stem cells from G0 to G(Alert). Nature 510: 393–6. 24870234 10.1038/nature13255 PMC4065227

[34] Kariya Y , Honma M , Aoki S , Chiba A , et al. 2009 Vps33a mediates RANKL storage in secretory lysosomes in osteoblastic cells. J Bone Miner Res 24: 1741–52. 19419298 10.1359/jbmr.090409

[35] Ye JH , Xu YJ , Gao J , Yan SG , et al. 2011 Critical‐size calvarial bone defects healing in a mouse model with silk scaffolds and SATB2‐modified iPSCs. Biomaterials 32: 5065–76. 21492931 10.1016/j.biomaterials.2011.03.053 PMC3100415

[36] Im JY , Min WK , You C , Kim HO , et al. 2013 Bone regeneration of mouse critical‐sized calvarial defects with human mesenchymal stem cells in scaffold. Lab Anim Res 29: 196–203. 24396384 10.5625/lar.2013.29.4.196 PMC3879338

