# Supplementary figures and images for: Peptide drugs accelerate BMP‐2‐induced calvarial bone regeneration and stimulate osteoblast differentiation through mTORC1 signaling
Source: Bioessays. 2016 Jun 27;38(8):717–25. doi: 10.1002/bies.201600104 (PMC5094554; doi:10.1002/bies.201600104)

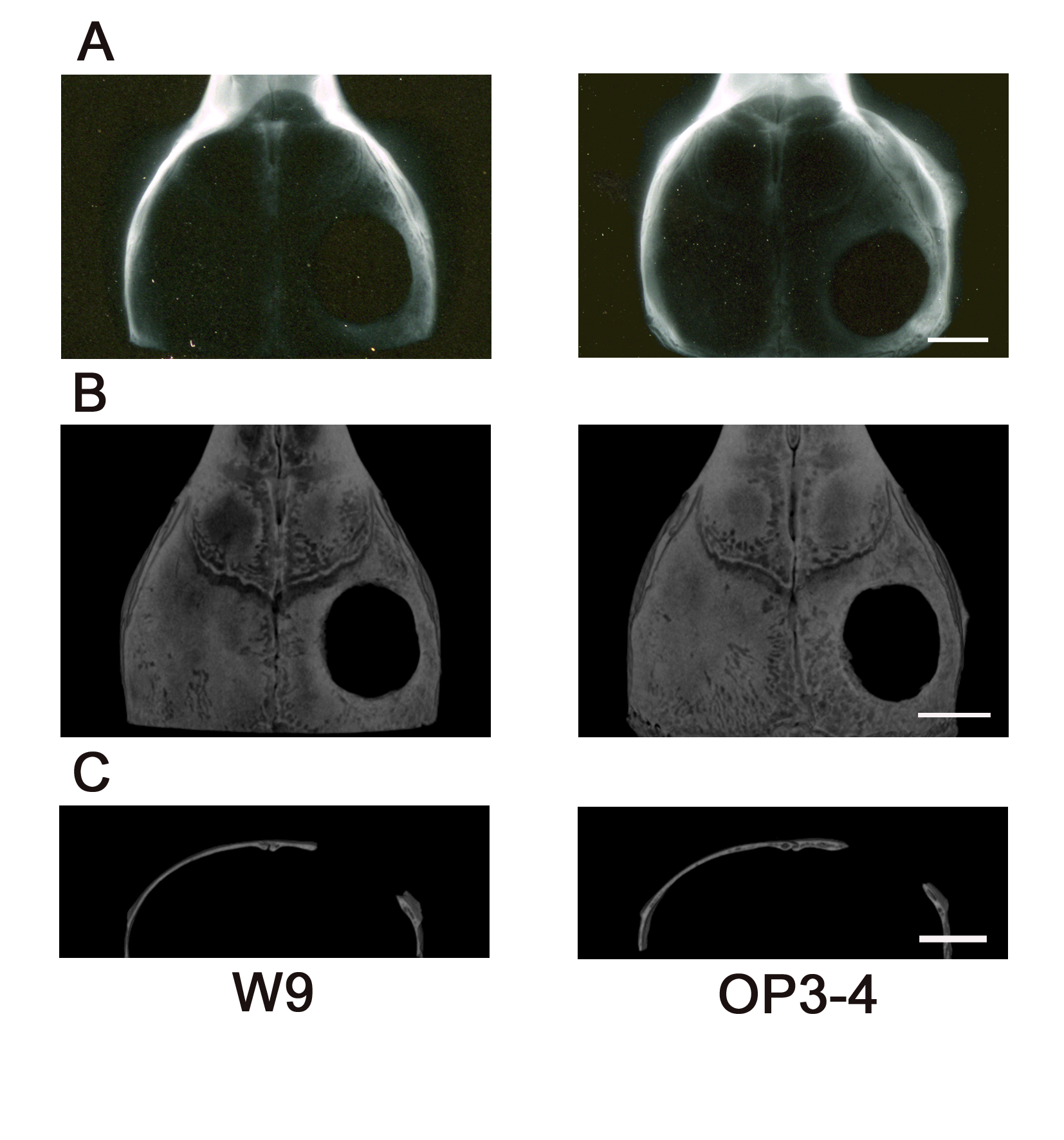

Supplement: Supplementary file 1 — Fig. S1. RANKL‐binding peptides without BMP‐2 did not promote bone regeneration in a murine calvarial defect model. [file BIES-38-717-s001.tif]

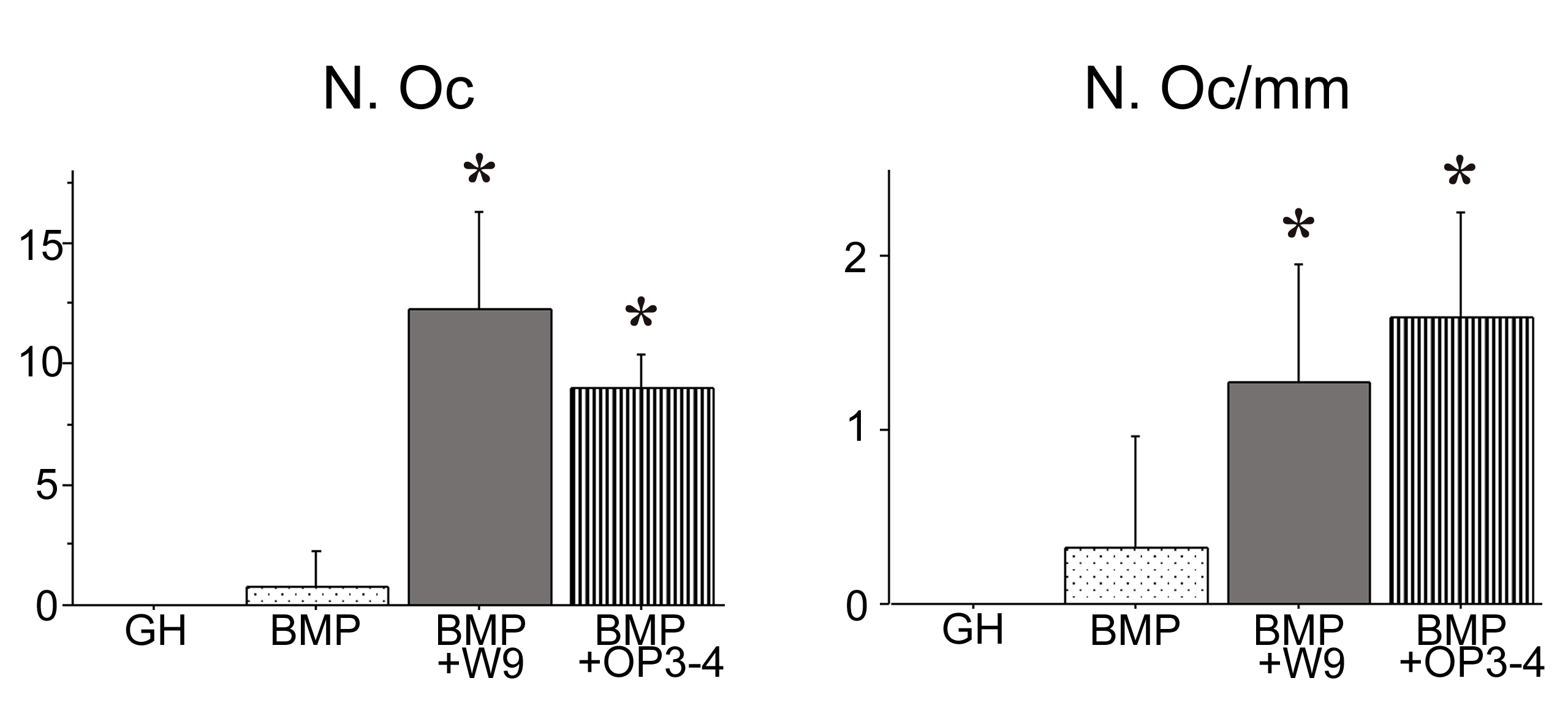

Supplement: Supplementary file 2 — Fig. S2. Bone resorption parameters at the bone regeneration site. [file BIES-38-717-s002.tif]
