# Supplementary material for: Peptide drugs accelerate BMP‐2‐induced calvarial bone regeneration and stimulate osteoblast differentiation through mTORC1 signaling
Source: Bioessays. 2016 Jun 27;38(8):717–25. doi: 10.1002/bies.201600104 (PMC5094554; doi:10.1002/bies.201600104)
Supplement: Supplementary file 3 — Supplementary Figure Legends [file BIES-38-717-s003.pdf]

Supplementary Methods

Histological preparation and counting the number of osteoclasts

Undecalcified frozen sections measuring 5 μm in thickness were prepared as described elsewhere [29]. TRAP staining and the measurement of the number of osteoclasts (N.Oc) and the bone perimeter was performed in a rectangular ROI (1.4 mm × 1.8 mm) at the center of the defect site, using a KS400 image analyzing system, as previously described [15,30].

Supplementary Figure Legends

Supplementary Fig. S1. RANKL-binding peptides without BMP-2 did not promote bone regeneration in a murine calvarial defect model. (A) Representative soft X-ray radiographic images of the calvariae treated with gelatin hydrogel containing W9 or OP3-4 for 28 days. (B, C) Representative μ-CT images of the whole mount of the calvariae (B) and a cross section at the defect site (C) are shown. All the scale bars represents 2 mm.

Supplementary Fig. S2. Bone resorption parameters at the bone regeneration site. The total number of osteoclasts at the region of interest in shown in the graph on the left. The graph on the right shows the number of osteoclasts per unit bone perimeter. Data are presented as the mean ± standard deviation (SD). *p <0.05 vs. BMP groups.
